# Influence of preoperative variables on the 3-month functional outcomes of the Vivity extended depth-of-focus intraocular lens: a prospective case series

**DOI:** 10.1186/s40662-024-00424-y

**Published:** 2025-02-02

**Authors:** Giacomo Savini, Alice Galzignato, Catarina P. Coutinho, Jinhai Huang, Yue Wu, Piero Barboni, João Mendanha Dias, Filomena J. Ribeiro, Domenico Schiano-Lomoriello

**Affiliations:** 1 I.R.C.C.S. - G.B. Bietti Foundation, Rome, Italy; 2 Studio Oculistico d’Azeglio, Bologna, Italy; 3 Università di Bologna, Dipartimento di Farmacia e Biotecnologie, Bologna, Italy; 4 Eye Institute and Department of Ophthalmology, Eye & ENT Hospital, Fudan University; NHC Key laboratory of Myopia and Related Eye Diseases; Key Laboratory of Myopia and Related Eye Diseases, Chinese Academy of Medical Sciences, Shanghai, China; 5 Shanghai Research Center of Ophthalmology and Optometry, Shanghai, China; 6 San Raffaele Hospital, Milan, Italy; 7 GoLP/IPFN, Instituto Superior Técnico, Universidade de Lisboa, Lisbon, Portugal; 8 Hospital da Luz Lisboa, Lisbon, Portugal; 9 Lisbon University, Lisbon, Portugal; 10 Visual Science Research Centre, Lisbon, Portugal

**Keywords:** Presbyopia, Intraocular lens, Cataract

## Abstract

**Background:**

To investigate the functional results of the AcrySof IQ Vivity (Alcon, Fort Worth, TX) extended depth-of-focus intraocular lens (EDoF-IOL) and explore correlations between the preoperative biometric parameters and the postoperative functional outcomes.

**Methods:**

In a prospective, single-center, non-randomized study, axial length, keratometry, anterior chamber depth, scotopic and photopic pupil diameters, pupil decentration, corneal asphericity, corneal higher-order aberrations (HOAs), coma and spherical aberration were measured preoperatively. The EDoF-IOL was implanted bilaterally. Three months postoperatively, manifest refraction, monocular and binocular uncorrected and corrected visual acuity at 4 m, 66 cm and 40 cm, binocular defocus curve, binocular contrast sensitivity, halometry and Strehl ratio were measured. Visual disturbances and spectacle independence were assessed with McAlinden and IOLSAT questionnaires, respectively. Assuming a minimum Pearson r correlation coefficient between variables of 0.5 with a power of 80% and a *P* value less than 0.05, a minimum sample size of 29 (58 eyes) cases was required.

**Results:**

Forty-three patients were enrolled. Binocular distance corrected visual acuity was lower than 0.1 logMAR for a defocus between + 1.0 and − 1.5 D. The mean values at 66 cm and 40 cm were − 0.07 ± 0.06 and 0.19 ± 0.13 logMAR, respectively. McAlinden’s questionnaire revealed mean scores close to zero for all questions. The IOLSAT questionnaire showed that spectacles were never used for distance and intermediate vision. Regression analysis did not disclose any significant correlation between the preoperatively measured variables and the postoperative outcomes, with a few exceptions: preoperative higher order corneal aberrations were correlated to halometry area (r^2^ = 0.2592, *P* = 0.0006) and the Q value to contrast sensitivity (r^2^ = 0.1717, *P* = 0.00574) under photopic conditions with glare at a spatial frequency of 18 cpd and without glare for all spatial frequencies (*P* < 0.01); it was also correlated to contrast sensitivity under mesopic conditions without glare at a spatial frequency of 12 cpd (r^2^ = 0.2311, *P* = 0.0011).

**Conclusions:**

In healthy unoperated eyes, the visual outcomes for this EDoF-IOL are independent of most of the patients’ preoperative parameters. Attention should be paid to preoperative corneal aberrations and asphericity, which did not lead to visual disturbances, but may be potential sources of halo and reduced contrast sensitivity.

## Background

The AcrySof IQ Vivity (Alcon, Fort Worth, TX) is a new extended depth-of-focus intraocular lens (EDoF-IOL) developed to provide patients with spectacle independence for far and intermediate vision while minimizing unwanted visual disturbances. It takes advantage of a novel EDoF-IOL optical technology known as wavefront shaping, which modifies the wavefront of the light to change its spatial propagation, producing a continuous extended range of vision from distance to functional near [1]. More specifically, it comprises a 2.2 mm diameter central zone and a peripheral ring that shows different aspheric profiles and works synergistically to generate a continuous EDoF. As a result, reconstructed wavefronts show a negative 4th-order spherical aberration and positive values from the 6th- to 14th-order spherical aberrations [2]. Previous studies have demonstrated that this lens provides superior intermediate and near visual acuity, along with non-inferior distance-corrected visual acuity (DCVA) compared to monofocal IOLs, with a similar visual disturbance profile [3–8]. In comparison with diffractive multifocal IOLs (MF-IOLs), the new EDoF-IOL has been shown to lead to a lower incidence of glare and halo [9, 10]. However, there is a lack of information regarding the potential influence of preoperative variables on the visual performance of this IOL and we cannot exclude that some of them, such as, for example, pupil diameter, corneal asphericity and angle kappa [11], may exert some effect.

The aim of this study was to investigate the functional results of this IOL and explore possible correlations between the postoperative functional outcomes and the preoperatively measured biometric parameters; we hope to understand whether the visual performance of this IOL can be preoperatively predicted by any variable. Knowing this information may be useful in the selection of candidates to the implantation of the AcrySof IQ Vivity, since patients with preoperative variables negatively affecting the postoperative performance may be excluded and offered different solutions. On the other hand, patients with preoperative variables improving postoperative performance (e.g., intermediate and near visual acuity) may be given higher expectations.

## Methods

This was a prospective, single-center, non-randomized case series. Consecutive patients undergoing bilateral implantation of the AcrySof IQ Vivity EDoF-IOL were enrolled. The study methods complied with the tenets of the Declaration of Helsinki for the use of human participants in biomedical research and were approved by the Comitato Etico di Area (No. 345–2021-DISP-AUSLBO). Informed consent was obtained from all patients.

Patients were included between January 2022 and December 2022 if they were 22 years of age or older at the time of surgery and had been diagnosed with bilateral cataract or required refractive lens exchange (RLE) to correct presbyopia. They had to be able to comprehend and willing to sign informed consent and complete all required postoperative follow-up procedures. The calculated lens power had to be within the available range.

The following preoperative exclusion criteria were adopted: clinically significant corneal abnormalities including corneal dystrophies and irregularities; severe dry eye with positive fluorescein staining of the ocular surface; previous corneal surgery; glaucoma and any disease or pathology, other than cataract, that was expected to reduce the potential postoperative DCVA to a level worse than 0.30 logMAR.

Patients were excluded in case of any intraoperative complications requiring further intervention (including but not limited to posterior capsule rupture, with vitreous loss, zonular dehiscence, which may make the IOL implant less stable, etc.), bag-sulcus, sulcus-sulcus or unknown placement of the haptics, any capsulorrhexis other than circular continuous capsulorrhexis (e.g., anterior radial inconsistencies in the capsulorrhexis such as anterior capsular tears or any areas of ‘can-opener’ capsulotomy).

### Preoperative measurements

Axial length, keratometry and anterior chamber depth (ACD, from the corneal epithelium to the anterior lens surface) were assessed by means of an optical biometer (IOLMaster 700, Zeiss, software version 1.80). Scotopic and photopic pupil diameters, pupil decentration (i.e., angle kappa), corneal asphericity (Q values at 8 mm), corneal higher-order aberrations (HOAs), coma and spherical aberration were measured with a rotating Scheimpflug camera combined with a Placido disc corneal topographer [Sirius, Costruzione Strumenti Oftalmici (CSO), software version 3.7]. Corneal aberrations were measured over a diameter corresponding to the photopic pupil diameter measured with the same device.

### Intraocular lens

The AcrySof IQ Vivity is an EDoF-IOL that uses wavefront shaping technology to stretch and shift the wavefront. The physical characteristics have been described in detail in previous reports [2–4]. Patients with an optimized keratometric astigmatism less than 0.75 diopters (D) received the non-toric model (DFT015), whereas those with an optimized keratometric astigmatism above this threshold were implanted with a toric model (DFT215, DFT315, DFT415, DFT515 and DFT615). Keratometric astigmatism optimization was carried out according to the method of Savini and colleagues [12]. IOL power was calculated with the Hoffer QST formula after constant optimization [13]. We did not select different IOL formulas for different axial length ranges since the Hoffer QST (like all new generation formulas) has been shown to be accurate in short, medium and long eyes [13]. The refractive target was zero for both eyes (the IOL power predicting the postoperative refraction closest to zero was selected), with no attempted mini-monovision.

### Surgical technique

Phacoemulsification was performed by the same surgeon (P.B.) through a temporal clear cornea 2.4-mm incision under topical anesthesia. The incision was always along the 180° meridian because all eyes (including those with a non toric IOL) underwent intraoperative digital marking. A continuous curvilinear capsulorrhexis with a diameter of approximately 5.0 mm was made. The IOL was implanted in the bag, and in the case of the toric models, the markings were oriented along the steep corneal meridian. An automated system (Verion, Alcon Laboratories, Inc.) was used to position the IOL markings on the intended axis of orientation. Surgery in the second eye was performed one week after surgery in the first eye.

### Postoperative examinations

Standard postoperative checks were performed on days 1, 4 and 30. Three months postoperatively, the following measurements were carried out in patients with binocular implantation of the EDoF-IOL:

1. Manifest refraction (MR) was obtained with a 4-m ETDRS chart and adjusted to infinity by adding − 0.25 D [14].
2. Monocular and binocular uncorrected distance visual acuity (UDVA) were assessed with an ETDRS chart at 4 m. Monocular and binocular CDVA were assessed with the same chart (for this purpose, the vergence induced by the presentation distance was corrected by adding + 0.25 D to the MR) [14].
3. Monocular and binocular distance-corrected intermediate visual acuity (DCIVA) and distance-corrected near visual acuity (DCNVA) were assessed with an ETDRS chart (Precision Vision) at 66 and 40 cm, respectively. All visual acuity measurements were recorded in logMAR and converted also into Snellen equivalent.
4. The binocular defocus curve (visual acuity over imposed defocus) was recorded under photopic conditions by adding negative lenses in 0.5 D steps up to − 4.5 D and positive lenses up to + 1.0 D to the distance-corrected MR [14, 15]. In this curve, the intermediate vision corresponds to − 1.5 and − 1.0 D defocus. The DCNVA corresponds to − 2.5 D defocus.
5. Binocular contrast sensitivity was measured with and without glare under photopic and mesopic conditions using the CSV-1000 HGT (Vector Vision, Inc.) with a chart distance of 2.45 m [16]. Under photopic conditions, contrast sensitivity was measured with and without glare at spatial frequencies of 3 cpd, 6 cpd, 12 cpd, and 18 cpd. Chart lighting was approximately 85 cd/m^2^, and the ambient light level was lower than chart luminance. Under mesopic conditions, contrast sensitivity was measured with and without glare at spatial frequencies of 3 cpd, 6 cpd, 12 cpd, and 18 cpd. Chart lighting was approximately 85 cd/m^2^, and subjects were fitted with neutral density filters to create appropriate subject-perceived chart luminance of approximately 3 cd/m^2^. Room lighting was dim to dark with the ambient lighting level lower than subject-perceived chart luminance. The glare source was adjusted to yield an illumination of approximately 2.5 cd/m^2^. Results were reported in log_10_ units (logCS) [14].
6. Halometry: objective assessment of visual halo was obtained with a validated halometer, which comprised a bright light-emitting diode (LED) glare source in the center of an iPad 4 [17]. Letters subtending 0.21 degrees (~ 0.3 logMAR) were moved centrifugally from the LED in 0.1 degree steps in eight orientations separated by 45 degrees with 500 Weber contrast units (C_w_). A working distance of 2 m was used as recommended by the developer (James SW Wolffsohn, personal communication).
7. Total ocular aberrometry: the Strehl ratio was assessed with an aberrometer (version 3.7, Osiris, CSO, Florence, Italy) based on a high-resolution four-faced pyramid wavefront sensor, which has been shown to provide repeatable measurements [18]. Measurements were performed in a dark room over a diameter corresponding to the scotopic pupil.
8. Visual disturbances questionnaire: patients were asked if they were suffering from visual disturbances at night, including glare, halos, starbursts, hazy vision, blurred vision, distortion and monocular double vision. We relied on the images and the scale described by McAlinden et al., where responses were graded on the basis of frequency, severity and bothersome scores along a scale ranging from 0 (no disturbances) to 3 (maximum disturbance) [19].
9. Spectacle independence questionnaire: the Italian version of the validated IOL Satisfaction (IOLSAT) questionnaire was used to assess the need for spectacles for far, intermediate and near vision under bright and dim light conditions. This questionnaire, which has been used by other authors [20], was developed by Alcon and recognized as validated based on guidance from the Food and Drug Administration. It has a score ranging from 0 (no need for spectacles) to 4 (spectacles always needed).

### Statistical analysis

Using Python 3.8 (Anaconda), each postoperative measurement was correlated with the preoperative variables and its statistical significance was determined. Due to the high number of correlations performed, a significance level of 1% was considered (*P* < 0.01). Since biometric measurements are highly correlated between the right and left eyes of the same individuals [21–24], binocular postoperative measurements (e.g., defocus curve or contrast sensitivity) were correlated with the average of the preoperative variable measured in the right and left eyes.

Assuming a minimum Pearson r correlation coefficient between variables of 0.5 with a power of 80% and a *P* < 0.05, a minimum sample size of 29 (58 eyes) cases was required (the power analysis was conducted in R version 4.2.2, with the pwr package). This sample size is similar to the datasets enrolled in recent noncomparative studies on EDOF-IOLs, where approximately 30 bilateral patients were included [8, 25–28].

## Results

Forty-three patients (mean age 65.10 ± 9.92 years; 25 females) were enrolled. Twenty-three patients (mean age 72.64 ± 6.92 years) underwent cataract surgery and 20 (mean age 56.80 ± 5.34 years) underwent RLE. None of them were lost to follow-up. The preoperative data of the 86 eyes are shown in Table 1. Some statistically significant differences were found between eyes with and without cataract: in the RLE subgroup the ACD was higher (3.19 ± 0.33 vs. 2.99 ± 0.32 mm, *P* = 0.006), the LT was thinner (4.48 ± 0.29 vs. 4.81 ± 0.27 mm, *P* < 0.0001), the Q value at 8 mm was less negative (− 0.17 ± 0.11 vs. − 0.27 ± 0.15, *P* = 0.0006) and lower values were measured for HOAs (0.13 ± 0.07 vs. 0.25 ± 0.22 μm, *P* = 0.0012), coma (0.05 ± 0.04 vs. 0.12 ± 0.15 μm, *P* = 0.0024) and spherical aberration (0.04 ± 0.10 vs. 0.12 ± 0.26 μm, *P* = 0.0181). A toric IOL was implanted in 53 eyes. No adverse events were reported. For each one of the parameters in Table 1, apart from the IOL power, no significant difference was found when comparing right and left eye measurements for the same patient.

**Table 1:** Preoperative measurements in the 43 patients who underwent Vivity IOL implantation

Parameters	Mean ± SD	Range	95% CI
Keratometry, flat meridian (D)	42.86 ± 1.32	39.54 to 46.54	[42.58, 43.14]
Keratometry, steep meridian (D)	43.69 ± 1.23	41.16 to 47.78	[43.42, 43.95]
Anterior chamber depth (mm)	3.08 ± 0.34	2.16 to 3.84	[3.01, 3.15]
Axial length (mm)	23.78 ± 1.26	21.63 to 27.37	[23.51, 24.04]
Q value (8 mm)	− 0.22 ± 0.14	− 0.77 to 0.09	[− 0.25, − 0.19]
Photopic pupil diameter (mm)	2.95 ± 0.63	1.67 to 4.50	[2.81, 3.08]
Scotopic pupil diameter (mm)	4.95 ± 0.89	2.59 to 6.80	[4.76, 5.13]
Pupil decentration (mm)	0.28 ± 0.16	0.02 to 1.15	[0.24, 0.31]
Corneal higher order aberrations (µm)	0.19 ± 0.18	0.04 to 0.99	[0.15, 0.23]
Corneal coma (µm)	0.09 ± 0.11	0.00 to 0.61	[0.06, 0.11]
Corneal spherical aberration (µm)	0.10 ± 0.21	0.00 to 1.42	[0.06, 0.14]
IOL power (D)	21.03 ± 3.99	10.00 to 28.00	[20.19, 21.87]

*SD* = standard deviation; *CI* = confidence interval; *IOL* = intraocular lens

Before MR was adjusted to infinity, the mean postoperative spherical equivalent was − 0.03 ± 0.19 D, with a range between − 0.50 and + 0.50 D. After adjustment to infinity, it decreased to − 0.28 ± 0.19 D. Measurements of DCVA, UDVA, DCIVA and DCNVA are shown in Table 2. They show excellent values for distance and intermediate vision and moderate outcomes for near vision, where a large variability can be observed. Ninety-four percent of eyes achieved monocular DCIVA of better than or equal to 0.2 logMAR (20/32). No differences were found between eyes that underwent cataract surgery and RLE, with the exception of monocular DCVA, which was better in the RLE subgroup (− 0.09 ± 0.04 vs. − 0.03 ± 0.08 logMAR, *P* < 0.0001) and monocular and binocular DCIVA, which were also better in the RLE subgroup (0.01 ± 0.10 vs. 0.11 ± 0.13 logMAR, *P* = 0.0002, and − 0.03 ± 0.08 vs. 0.04 ± 0.07 logMAR, *P* = 0.008, respectively, Tables 3, 4, 5).

**Table 2:** Monocular and binocular postoperative values of visual acuity. All values are expressed as logMAR and Snellen

Parameters	Mean ± SD LogMAR Snellen	Range LogMAR Snellen	95% CI
Monocular UCDVA	− 0.02 ± 0.11 20/19	− 0.10, + 0.63 20/32, 20/16	[− 0.04, 0.00]
Binocular UCDVA	− 0.08 ± 0.05 20/17	− 0.20, + 0.00 20/27, 20/13	[− 0.09, − 0.07]
Monocular DCVA	− 0.06 ± 0.07 20/18	− 0.10, + 0.20 20/32, 20/16	[− 0.07, − 0.04]
Binocular DCVA	− 0.07 ± 0.06 20/17	− 0.20, + 0.00 20/25, 20/13	[− 0.08, − 0.06]
Monocular DCIVA	0.06 ± 0.13 20/23	− 0.20, + 0.50 20/63, 20/13	[0.04, 0.09]
Binocular DCIVA	0.01 ± 0.08 20/20	− 0.20, + 0.20 20/32, 20/13	[− 0.01, 0.03]
Monocular DCNVA	0.30 ± 0.16 20/40	0.00, + 1.00 20/200, 20/20	[0.27, 0.34]
Binocular DCNVA	0.19 ± 0.13 20/31	− 0.10, + 0.60 20/80, 20/16	[0.17, 0.22]

*SD* = standard deviation; *CI *= confidence interval; *UCDVA* = uncorrected distance visual acuity; *DCVA* = distance-corrected visual acuity; *DCIVA* = distance-corrected intermediate visual acuity; *DCNVA* = distance-corrected near visual acuity

**Fig. 1:**
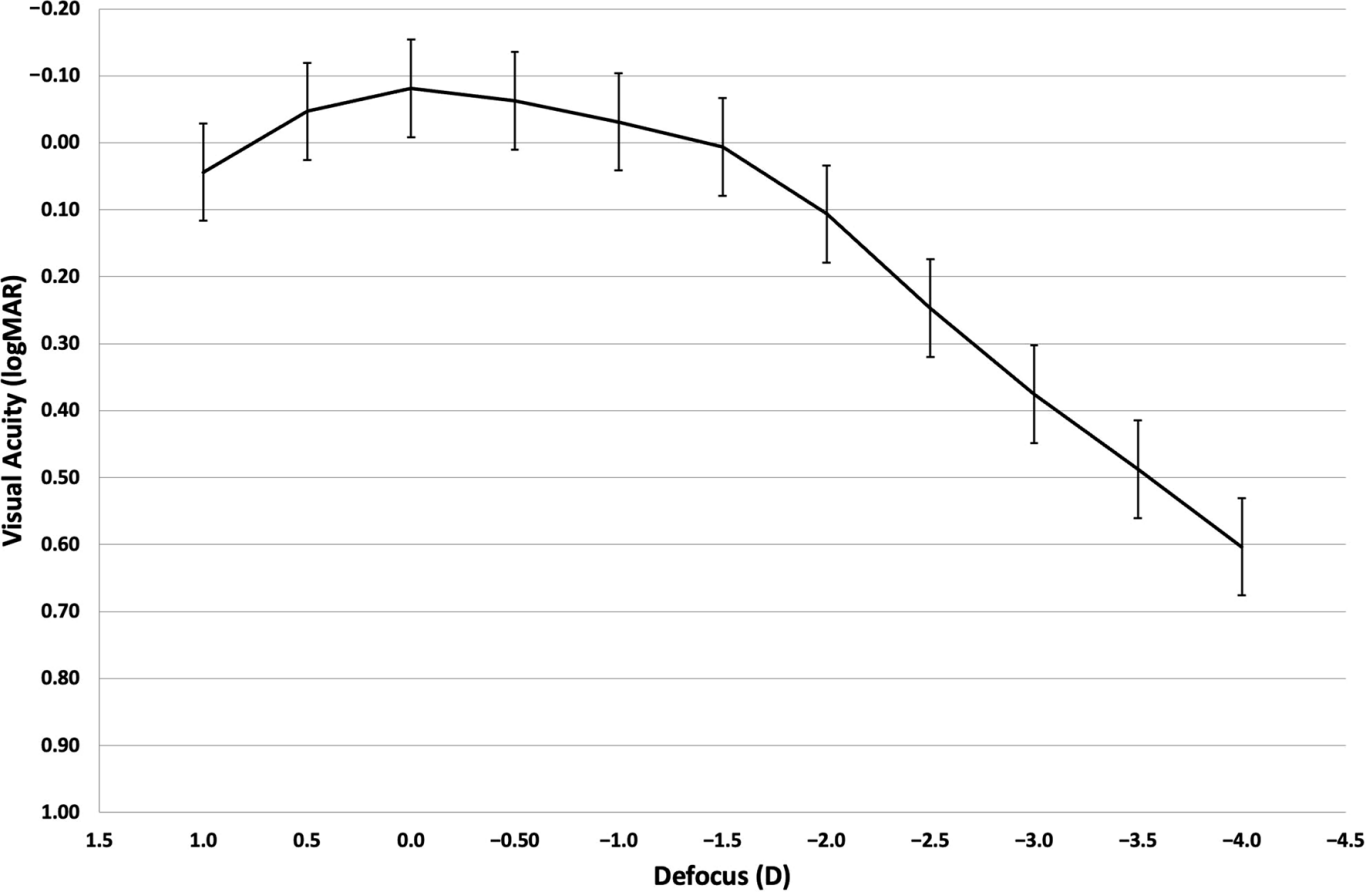
Binocular defocus curve for the whole sample (recorded with distance-corrected manifest refraction). Error bars describe the standard deviation

**Fig. 2:**
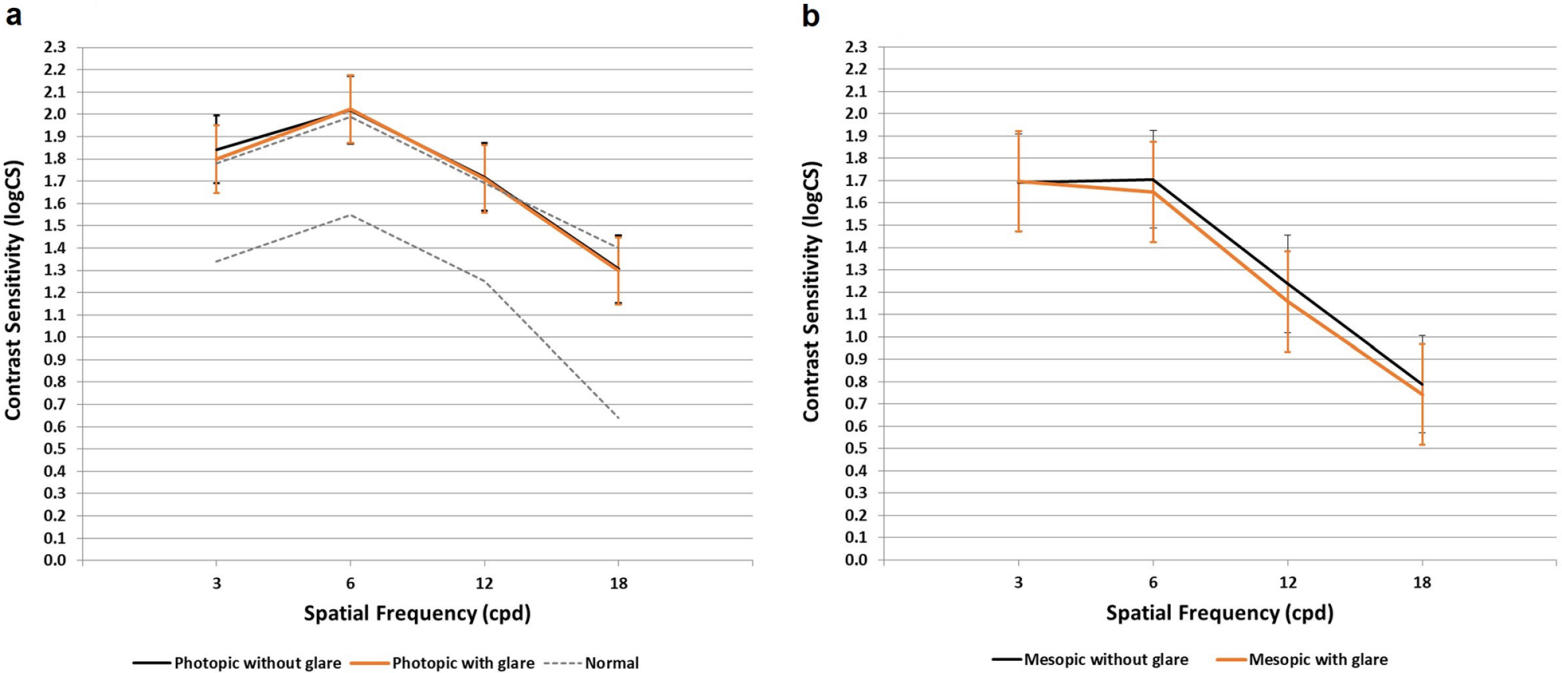
Binocular photopic (**a**) and mesopic (**b**) contrast sensitivity, with and without glare. The dashed line represents the normal range according to the manufacturer of the instrument used to measure the contrast sensitivity for the photopic condition

**Fig. 3:**
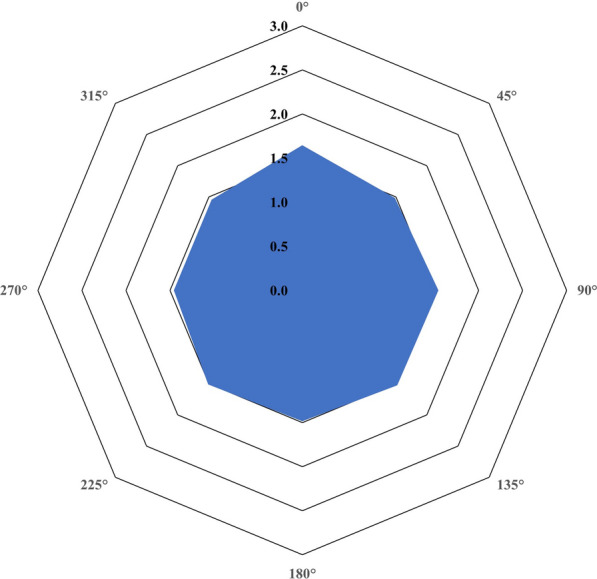
Results of the Wolffsohn halometer

The Strehl ratio was 0.34 ± 0.17 with a range between 0.10 and 0.81. The mean value was higher in the RLE subgroup (0.40 ± 0.19 vs. 0.30 ± 0.14, *P* = 0.0066). Figures 1, 2 and 3 show the results of the binocular defocus curve, binocular contrast sensitivity and binocular halometry, respectively. According to the binocular defocus curve, DCVA was lower than 0.1 logMAR (higher than 20/25) for a defocus between + 1.0 and − 1.5 D; DCVA progressively decreased once the defocus was set at − 2.0 D. Statistically significant differences were observed in the cataract and RLE subgroups, since the latter displayed better mean values with a defocus of − 1.0 D (− 0.07 ± 0.05 vs. − 0.02 ± 0.06 logMAR, *P* = 0.0140), − 1.5 D (− 0.03 ± 0.07 vs. 0.01 ± 0.07 logMAR, *P* = 0.0439) and − 2.0 D (0.06 ± 0.08 vs. 0.13 ± 0.13 logMAR, *P* = 0.0364). Figure 2 shows higher values for photopic than mesopic contrast sensitivity, with almost no difference with and without glare. Photopic contrast sensitivity outcomes are within the normal range. For all values of photopic and mesopic contrast sensitivity, the RLE subgroup revealed better outcomes (*P* values ranging between 0.0146 and < 0.0001), with the exception of mesopic contrast sensitivity without glare at a frequency of 3 cpd. Figure 3 shows a limited area for the postoperatively measured halo; in this regard, the mean values of patients that underwent RLE showed a lower area in all orientations (*P* < 0.0001).

McAlinden’s questionnaire revealed mean scores close to zero for all questions, and thus demonstrate that this IOL does not induce relevant visual disturbances. The worst outcomes were observed for halo, which revealed a mean frequency of 0.23 ± 0.61 (range: 0 to 2), a mean severity of 0.14 ± 0.46 (range: 0 to 2) and a mean bothersome score of 0.21 ± 0.60 (range: 0 to 2). A couple of cases (n = 2) of mild disturbances were reported for glare and starbursts, but not for blurred vision, distortion or double vision. No statistically significant differences were detected between cataract and RLE groups.

The IOLSAT questionnaire showed that spectacles were never used for distance and intermediate vision under high and low light conditions (mean score: 0.00 ± 0.00). On average, spectacles were rarely required for near vision under high light conditions (mean score: 0.98 ± 1.29, range: 0 to 4) and sometimes for near vision under low light conditions (mean score:1.74 ± 1.60, range: 0 to 4).

Regression analysis did not reveal any significant correlation between the preoperatively measured variables and the postoperative outcomes (Tables 3, 4 and 5), with a few exceptions. Namely, the preoperative corneal aberrations were correlated to the postoperative halometry area: HOA showed high r (0.5091) and r^2^ (0.2592) values, giving *P* = 0.000575. Significant correlations were also detected for coma (r = 0.4656, r^2^ = 0.2168, *P* = 0.00189) and spherical aberration (r = 0.5251, r^2^ = 0.2757, *P* = 0.000356). These correlations were no longer statistically significant when the two subgroups were analyzed separately. Furthermore, a statistically significant correlation was found between the Q value at 8 mm and contrast sensitivity: under photopic conditions with glare at a spatial frequency of 18 cpd (r = 0.4143, r^2^ = 0.1717, *P* = 0.00574), and without glare for all spatial frequencies (*P* < 0.01); these correlations were maintained only in the cataract subgroup. Under mesopic conditions without glare, the correlation was significant at a spatial frequency of 12 cpd for the whole sample (r = 0.4807, r^2^ = 0.2311, *P* = 0.0011) but not for the two subsamples.

**Table 3:** Correlation coefficients and respective *P* values between preoperative measurements and postoperative DCVA (4 m/66 cm/40 cm), Strehl ratio and Wollsohn halometer area measurements

Parameters	DCVA 4 m	DCVA 66 cm	DCVA 40 cm	Strehl ratio	Wollsohn halometer area
KFlat (D)	r = − 0.211 r^2^ = 0.045 *P* = 0.174	r = 0.104 r^2^ = 0.011 *P* = 0.505	r = 0.006 r^2^ = 0.000 *P* = 0.969	r = − 0.071 r^2^ = 0.005 *P* = 0.669	r = 0.129 r^2^ = 0.017 *P* = 0.416
KSteep (D)	r = − 0.110 r^2^ = 0.012 *P* = 0.483	r = 0.183 r^2^ = 0.033 *P* = 0.241	r = − 0.013 r^2^ = 0.000 *P* = 0.936	r = − 0.178 r^2^ = 0.032 *P* = 0.278	r = 0.165 r^2^ = 0.027 *P* = 0.298
ACD (mm)	r = − 0.329 r^2^ = 0.108 *P* = 0.031	r = − 0.178 r^2^ = 0.032 *P* = 0.254	r = − 0.170 r^2^ = 0.029 *P* = 0.275	r = 0.421 r^2^ = 0.178 *P* = 0.008	r = − 0.269 r^2^ = 0.072 *P* = 0.085
LT (mm)	r = 0.090 r^2^ = 0.008 *P* = 0.567	r = 0.180 r^2^ = 0.032 *P* = 0.248	r = 0.249 r^2^ = 0.062 *P* = 0.108	r = − 0.217 r^2^ = 0.047 *P* = 0.185	r = 0.280 r^2^ = 0.078 *P* = 0.072
AL (mm)	r = − 0.051 r^2^ = 0.003 *P* = 0.748	r = 0.000 r^2^ = 0.000 *P* = 0.999	r = − 0.073 r^2^ = 0.005 *P* = 0.643	r = 0.235 r^2^ = 0.055 *P* = 0.151	r = 0.020 r^2^ = 0.000 *P* = 0.902
Photopic PD (mm)	r = 0.078 r^2^ = 0.006 *P* = 0.631	r = 0.180 r^2^ = 0.032 *P* = 0.267	r = 0.075 r^2^ = 0.006 *P* = 0.647	r = 0.378 r^2^ = 0.143 *P* = 0.021	r = − 0.252 r^2^ = 0.064 *P* = 0.121
Scotopic PD (mm)	r = 0.071 r^2^ = 0.005 *P* = 0.662	r = 0.115 r^2^ = 0.013 *P* = 0.480	r = − 0.064 r^2^ = 0.004 *P* = 0.696	r = 0.013 r^2^ = 0.000 *P* = 0.942	r = − 0.162 r^2^ = 0.026 *P* = 0.326
Pupil decentration (mm)	r = − 0.036 r^2^ = 0.001 *P* = 0.817	r = 0.019 r^2^ = 0.000 *P* = 0.903	r = 0.206 r^2^ = 0.042 *P* = 0.186	r = − 0.008 r^2^ = 0.000 *P* = 0.962	r = − 0.040 r^2^ = 0.002 *P* = 0.802
Q value, 8 mm	r = 0.084 r^2^ = 0.007 *P* = 0.591	r = − 0.058 r^2^ = 0.003 *P* = 0.713	r = 0.218 r^2^ = 0.048 *P* = 0.160	r = 0.250 r^2^ = 0.062 *P* = 0.125	r = − 0.312 r^2^ = 0.097 *P* = 0.045
HOA (µm)	r = − 0.128 r^2^ = 0.016 *P* = 0.414	r = 0.061 r^2^ = 0.004 *P* = 0.699	r = 0.057 r^2^ = 0.003 *P* = 0.718	r = − 0.010 r^2^ = 0.000 *P* = 0.954	r = 0.509 r^2^ = 0.259 *P* < 0.001
Coma (µm)	r = − 0.150 r^2^ = 0.022 *P* = 0.338	r = 0.064 r^2^ = 0.004 *P* = 0.683	r = 0.137 r^2^ = 0.019 *P* = 0.380	r = − 0.026 r^2^ = 0.001 *P* = 0.876	r = 0.466 r^2^ = 0.217 *P* = 0.002
SA (µm)	r = 0.108 r^2^ = 0.012 *P* = 0.490	r = 0.259 r^2^ = 0.067 *P* = 0.094	r = 0.325 r^2^ = 0.106 *P* = 0.033	r = − 0.225 r^2^ = 0.051 *P* = 0.169	r = 0.525 r^2^ = 0.276 *P* < 0.001

Due to the high number of correlations performed, a significance level of 1% was considered (*P* < 0.01)

*DCVA* = distance corrected visual acuity; *D* = diopters; *Kflat* = flat keratometry; *Ksteep* = steep keratometry; *ACD* = anterior chamber depth; *LT* = lens thickness; *AL* = axial length; *PD* = pupil diameter; *HOA* = higher order aberrations; *SA* = spherical aberration

**Table 4:** Correlation coefficients and respective *P* values between preoperative measurements and postoperative photopic contrast sensitivity with and without glare

Parameters	Photopic contrast sensitivity with glare				Photopic contrast sensitivity without glare			
	3 cpd	6 cpd	12 cpd	18 cpd	3 cpd	6 cpd	12 cpd	18 cpd
KFlat (D)	r = − 0.205 r^2^ = 0.042 *P* = 0.187	r = − 0.191 r^2^ = 0.037 *P* = 0.219	r = − 0.131 r^2^ = 0.017 *P* = 0.404	r = − 0.132 r^2^ = 0.018 *P* = 0.397	r = − 0.231 r^2^ = 0.054 *P* = 0.136	r = − 0.153 r^2^ = 0.023 *P* = 0.327	r = − 0.121 r^2^ = 0.015 *P* = 0.439	r = − 0.085 r^2^ = 0.007 *P* = 0.589
KSteep (D)	r = − 0.254 r^2^ = 0.065 *P* = 0.100	r = − 0.264 r^2^ = 0.070 *P* = 0.087	r = − 0.234 r^2^ = 0.055 *P* = 0.131	r = − 0.232 r^2^ = 0.054 *P* = 0.135	r = − 0.345 r^2^ = 0.119 *P* = 0.023	r = − 0.260 r^2^ = 0.068 *P* = 0.092	r = − 0.229 r^2^ = 0.053 *P* = 0.139	r = − 0.220 r^2^ = 0.049 *P* = 0.156
ACD (mm)	r = − 0.077 r^2^ = 0.006 *P* = 0.626	r = − 0.013 r^2^ = 0.000 *P* = 0.939	r = 0.158 r^2^ = 0.025 *P* = 0.312	r = 0.269 r^2^ = 0.072 *P* = 0.082	r = 0.179 r^2^ = 0.032 *P* = 0.250	r = 0.355 r^2^ = 0.126 *P* = 0.020	r = 0.204 r^2^ = 0.042 *P* = 0.189	r = 0.323 r^2^ = 0.105 *P* = 0.035
LT (mm)	r = − 0.251 r^2^ = 0.063 *P* = 0.105	r = − 0.214 r^2^ = 0.046 *P* = 0.167	r = − 0.226 r^2^ = 0.051 *P* = 0.146	r = − 0.274 r^2^ = 0.075 *P* = 0.076	r = − 0.313 r^2^ = 0.098 *P* = 0.041	r = − 0.372 r^2^ = 0.139 *P* = 0.014	r = − 0.254 r^2^ = 0.064 *P* = 0.101	r = − 0.333 r^2^ = 0.111 *P* = 0.029
AL (mm)	r = − 0.039 r^2^ = 0.002 *P* = 0.806	r = − 0.001 r^2^ = 0.000 *P* = 0.994	r = 0.109 r^2^ = 0.012 *P* = 0.487	r = 0.133 r^2^ = 0.018 *P* = 0.396	r = − 0.021 r^2^ = 0.004 *P* = 0.896	r = 0.079 r^2^ = 0.006 *P* = 0.613	r = 0.048 r^2^ = 0.002 *P* = 0.761	r = 0.126 r^2^ = 0.016 *P* = 0.42
Photopic PD (mm)	r = 0.147 r^2^ = 0.022 *P* = 0.366	r = 0.192 r^2^ = 0.037 *P* = 0.236	r = − 0.006 r^2^ = 0.000 *P* = 0.973	r = − 0.005 r^2^ = 0.000 *P* = 0.975	r = 0.148 r^2^ = 0.022 *P* = 0.363	r = 0.210 r^2^ = 0.044 *P* = 0.194	r = 0.193 r^2^ = 0.037 *P* = 0.234	r = 0.117 r^2^ = 0.014 *P* = 0.472
Scotopic PD (mm)	r = 0.015 r^2^ = 0.000 *P* = 0.927	r = 0.036 r^2^ = 0.001 *P* = 0.828	r = 0.022 r^2^ = 0.001 *P* = 0.892	r = − 0.023 r^2^ = 0.001 *P* = 0.887	r = 0.068 r^2^ = 0.005 *P* = 0.676	r = 0.050 r^2^ = 0.003 *P* = 0.758	r = 0.189 r^2^ = 0.036 *P* = 0.242	r = 0.059 r^2^ = 0.003 *P* = 0.720
Pupil decentration (mm)	r = 0.135 r^2^ = 0.018 *P* = 0.390	r = 0.080 r^2^ = 0.006 *P* = 0.610	r = 0.047 r^2^ = 0.002 *P* = 0.767	r = 0.094 r^2^ = 0.009 *P* = 0.549	r = 0.040 r^2^ = 0.000 *P* = 0.916	r = 0.050 r^2^ = 0.001 *P* = 0.885	r = 0.142 r^2^ = 0.006 *P* = 0.625	r = 0.144 r^2^ = 0.003 *P* = 0.730
Q value, 8 mm	r = 0.311 r^2^ = 0.097 *P* = 0.042	r = 0.334 r^2^ = 0.112 *P* = 0.029	r = 0.348 r^2^ = 0.121 *P* = 0.022	r = 0.414 r^2^ = 0.172 *P* = 0.006	r = 0.409 r^2^ = 0.167 *P* = 0.007	r = 0.436 r^2^ = 0.190 *P* = 0.004	r = 0.430 r^2^ = 0.185 *P* = 0.004	r = 0.462 r^2^ = 0.214 *P* = 0.002
HOA (µm)	r = − 0.062 r^2^ = 0.004 *P* = 0.692	r = 0.057 r^2^ = 0.003 *P* = 0.716	r = − 0.14 r^2^ = 0.011 *P* = 0.506	r = − 0.222 r^2^ = 0.049 *P* = 0.152	r = − 0.100 r^2^ = 0.010 *P* = 0.534	r = − 0.131 r^2^ = 0.017 *P* = 0.401	r = − 0.053 r^2^ = 0.003 *P* = 0.736	r = − 0.231 r^2^ = 0.053 *P* = 0.136
Coma (µm)	r = − 0.045 r^2^ = 0.002 *P* = 0.773	r = 0.097 r^2^ = 0.009 *P* = 0.536	r = − 0.107 r^2^ = 0.011 *P* = 0.496	r = − 0.145 r^2^ = 0.021 *P* = 0.354	r = − 0.098 r^2^ = 0.010 *P* = 0.524	r = − 0.074 r^2^ = 0.005 *P* = 0.639	r = − 0.027 r^2^ = 0.001 *P* = 0.863	r = − 0.148 r^2^ = 0.020 *P* = 0.342
SA (µm)	r = 0.097 r^2^ = 0.009 *P* = 0.537	r = 0.173 r^2^ = 0.030 *P* = 0.269	r = − 0.119 r^2^ = 0.014 *P* = 0.446	r = − 0.188 r^2^ = 0.036 *P* = 0.226	r = − 0.076 r^2^ = 0.006 *P* = 0.629	r = 0.035 r^2^ = 0.001 *P* = 0.822	r = − 0.072 r^2^ = 0.005 *P* = 0.647	r = − 0.180 r^2^ = 0.032 *P* = 0.249

Due to the high number of correlations performed, a significance level of 1% was considered (*P* < 0.01)

*D* = diopters; *Kflat* = flat keratometry; *Ksteep* = steep keratometry; *ACD* = anterior chamber depth; *LT* = lens thickness; *AL* = axial length; *PD* = pupil diameter; *HOA* = higher order aberrations; *SA* = spherical aberration

**Table 5:** Correlation coefficients and respective *P* values between preoperative measurements and postoperative mesopic contrast sensitivity with and without glare

Parameters	Mesopic contrast sensitivity with glare				Mesopic contrast sensitivity without glare			
	3 cpd	6 cpd	12 cpd	18 cpd	3 cpd	6 cpd	12 cpd	18 cpd
KFlat (D)	r = − 0.030 r^2^ = 0.001 *P* = 0.848	r = − 0.211 r^2^ = 0.044 *P* = 0.175	r = − 0.075 r^2^ = 0.006 *P* = 0.631	r = − 0.110 r^2^ = 0.012 *P* = 0.482	r = − 0.128 r^2^ = 0.016 *P* = 0.414	r = 0.005 r^2^ = 0.000 *P* = 0.974	r = − 0.301 r^2^ = 0.091 *P* = 0.050	r = − 0.261 r^2^ = 0.068 *P* = 0.091
KSteep (D)	r = − 0.093 r^2^ = 0.009 *P* = 0.552	r = − 0.347 r^2^ = 0.121 *P* = 0.023	r = − 0.176 r^2^ = 0.031 *P* = 0.259	r = − 0.206 r^2^ = 0.042 *P* = 0.185	r = − 0.151 r^2^ = 0.028 *P* = 0.335	r = − 0.081 r^2^ = 0.007 *P* = 0.604	r = − 0.382 r^2^ = 0.146 *P* = 0.011	r = − 0.338 r^2^ = 0.115 *P* = 0.026
ACD (mm)	r = − 0.194 r^2^ = 0.038 *P* = 0.213	r = 0.081 r^2^ = 0.007 *P* = 0.607	r = 0.177 r^2^ = 0.031 *P* = 0.256	r = 0.094 r^2^ = 0.009 *P* = 0.551	r = − 0.091 r^2^ = 0.008 *P* = 0.561	r = 0.323 r^2^ = 0.104 *P* = 0.035	r = 0.282 r^2^ = 0.079 *P* = 0.067	r = 0.049 r^2^ = 0.002 *P* = 0.755
LT (mm)	r = − 0.202 r^2^ = 0.041 *P* = 0.194	r = − 0.248 r^2^ = 0.062 *P* = 0.108	r = − 0.301 r^2^ = 0.091 *P* = 0.05	r = − 0.174 r^2^ = 0.030 *P* = 0.264	r = − 0.030 r^2^ = 0.001 *P* = 0.834	r = − 0.504 r^2^ = 0.254 *P* < 0.001	r = − 0.435 r^2^ = 0.189 *P* = 0.004	r = − 0.236 r^2^ = 0.056 *P* = 0.128
AL (mm)	r = − 0.084 r^2^ = 0.007 *P* = 0.592	r = − 0.090 r^2^ = 0.008 *P* = 0.565	r = 0.084 r^2^ = 0.007 *P* = 0.592	r = 0.171 r^2^ = 0.029 *P* = 0.272	r = 0.097 r^2^ = 0.010 *P* = 0.535	r = 0.078 r^2^ = 0.006 *P* = 0.617	r = 0.219 r^2^ = 0.048 *P* = 0.158	r = 0.221 r^2^ = 0.050 *P* = 0.154
Photopic PD (mm)	r = 0.154 r^2^ = 0.024 *P* = 0.344	r = − 0.003 r^2^ = 0.000 *P* = 0.986	r = 0.093 r^2^ = 0.009 *P* = 0.567	r = 0.070 r^2^ = 0.005 *P* = 0.669	r = 0.014 r^2^ = 0.000 *P* = 0.930	r = 0.103 r^2^ = 0.011 *P* = 0.527	r = 0.062 r^2^ = 0.004 *P* = 0.704	r = 0.091 r^2^ = 0.008 *P* = 0.577
Scotopic PD (mm)	r = 0.130 r^2^ = 0.017 *P* = 0.423	r = − 0.118 r^2^ = 0.014 *P* = 0.469	r = − 0.121 r^2^ = 0.015 *P* = 0.457	r = − 0.130 r^2^ = 0.017 *P* = 0.424	r = 0.046 r^2^ = 0.002 *P* = 0.780	r = 0.017 r^2^ = 0.000 *P* = 0.917	r = − 0.215 r^2^ = 0.016 *P* = 0.444	r = − 0.020 r^2^ = 0.000 *P* = 0.904
Pupil decentration (mm)	r = 0.093 r^2^ = 0.009 *P* = 0.554	r = − 0.004 r^2^ = 0.000 *P* = 0.981	r = − 0.010 r^2^ = 0.000 *P* = 0.952	r = 0.104 r^2^ = 0.011 *P* = 0.508	r = 0.222 r^2^ = 0.050 *P* = 0.152	r = 0.014 r^2^ = 0.000 *P* = 0.932	r = 0.143 r^2^ = 0.020 *P* = 0.362	r = 0.193 r^2^ = 0.037 *P* = 0.214
Q value, 8 mm	r = 0.111 r^2^ = 0.012 *P* = 0.481	r = 0.267 r^2^ = 0.071 *P* = 0.084	r = 0.377 r^2^ = 0.142 *P* = 0.013	r = 0.315 r^2^ = 0.099 *P* = 0.040	r = 0.129 r^2^ = 0.017 *P* = 0.411	r = 0.330 r^2^ = 0.109 *P* = 0.030	r = 0.481 r^2^ = 0.231 *P* = 0.001	r = 0.213 r^2^ = 0.045 *P* = 0.170
HOA (µm)	r = 0.009 r^2^ = 0.000 *P* = 0.953	r = 0.015 r^2^ = 0.000 *P* = 0.927	r = 0.006 r^2^ = 0.000 *P* = 0.968	r = − 0.153 r^2^ = 0.023 *P* = 0.327	r = 0.005 r^2^ = 0.000 *P* = 0.973	r = − 0.116 r^2^ = 0.013 *P* = 0.460	r = − 0.063 r^2^ = 0.004 *P* = 0.689	r = − 0.141 r^2^ = 0.020 *P* = 0.368
Coma (µm)	r = 0.035 r^2^ = 0.001 *P* = 0.826	r = − 0.004 r^2^ = 0.000 *P* = 0.981	r = − 0.018 r^2^ = 0.000 *P* = 0.907	r = − 0.161 r^2^ = 0.026 *P* = 0.304	r = 0.080 r^2^ = 0.006 *P* = 0.612	r = − 0.144 r^2^ = 0.201 *P* = 0.356	r = − 0.076 r^2^ = 0.006 *P* = 0.629	r = − 0.110 r^2^ = 0.012 *P* = 0.484
SA (µm)	r = 0.189 r^2^ = 0.036 *P* = 0.225	r = 0.004 r^2^ = 0.000 *P* = 0.981	r = 0.129 r^2^ = 0.017 *P* = 0.409	r = − 0.007 r^2^ = 0.000 *P* = 0.965	r = 0.204 r^2^ = 0.042 *P* = 0.189	r = − 0.236 r^2^ = 0.056 *P* = 0.127	r = 0.102 r^2^ = 0.010 *P* = 0.515	r = 0.013 r^2^ = 0.000 *P* = 0.936

Due to the high number of correlations performed, a significance level of 1% was considered (*P* < 0.01)

*D* = diopters; *Kflat* = flat keratometry; *Ksteep* = steep keratometry; *ACD* = anterior chamber depth; *LT* = lens thickness; *AL* = axial length; *PD* = pupil diameter; *HOA* = higher-order aberrations; *SA* = spherical aberration

## Discussion

The analyses conducted in this study indicate that patients with a bilaterally implanted AcrySof IQ Vivity IOL are satisfied with the refractive outcomes, as they achieved a good visual performance at both far and intermediate distances, as well as spectacle independence. The binocular defocus curves for visual acuity and contrast sensitivity under different conditions were found to be within the normal range expectable for an EDoF-IOL. No relevant visual disturbances at night were reported, even regarding halo, as the McAlinden questionnaire scores were close to zero. Overall, these findings are not new and are in good agreement with several studies that previously investigated this EDoF-IOL [3–7, 9, 10, 25]. The only new information was the difference observed between patients who underwent cataract surgery and those that underwent RLE. The latter, who were younger, demonstrated higher monocular DCVA, monocular and binocular DCIVA, better contrast sensitivity and halometry. These findings, which need to be validated by larger studies, may be related to several age-related changes of the tear film, corneal transparency and macular function.

Our aim was to extend the analysis by exploring potential correlations between postoperative functional outcomes and preoperatively measured biometric parameters. This was done to determine whether the visual performance of this IOL can be preoperatively predicted by any specific variable. The lack of correlations would suggest that, in healthy eyes, this EDoF-IOL may be implanted with a less strict selection criteria and would therefore be indicated in most patients. On the other hand, should any postoperative functional outcome be correlated to one or more preoperative variable, careful attention should be paid during preoperative examinations and counseling, and it might be necessary to warn some patients that the IOL is contraindicated due to the higher risk of unsatisfactory functional outcomes. In this regard, we should remember that—before EDoF-IOLs were introduced—the ASCRS Cataract Clinical Committee stated that “patient selection is one of the most challenging aspects of multifocal IOL use, being more art than science” [29]. We aimed to provide a higher degree of scientific evidence for patient selection, given that still today there is no unanimous consensus among experts about the criteria for recommending a presbyopia-correcting IOL [30].

The correlation analysis overall revealed no significant relationship between the preoperative and postoperative parameters, indicating that this IOL can be safely implanted in eyes with healthy corneas and no other pathologies. From a statistical point of view, two exceptions were detected. First, we observed that higher values of preoperative corneal aberrations (HOA, coma, spherical aberration) were significantly correlated to the postoperative halometry. This finding is not surprising as an increase in HOAs has been known to negatively impact visual function [31]. Accordingly, the ESASO Study Group agreed that corneal HOAs should not exceed a given threshold when selecting candidates for presbyopia-correcting IOLs [30]. However, notwithstanding the positive correlation between corneal aberrations and postoperative halometry, no patients in our sample complained about visual disturbances, suggesting that even higher values of halo were not sufficient to induce any subjective complaint. The second exception was related to preoperative corneal asphericity; more negative Qvalues were significantly correlated to lower contrast sensitivity under both photopic and mesopic conditions. This is not surprising, since corneal asphericity is directly correlated to spherical aberration [31], which is known to deteriorate contrast sensitivity [32, 34].

Interestingly, neither photopic nor scotopic pupil size revealed any statistically significant correlation with the postoperative functional outcomes. An in vitro study by Fernàndez-Vega-Cueto et al. showed that the optical performance of this IOL is influenced by the pupil size – a 3-mm pupil reduces the optical quality compared to a 4.5-mm pupil [8]. However, our data suggest that such a reduction in optical quality does not lead to any clinically relevant decrease in visual performance in a large range of photopic (1.67 to 4.50 mm) and scotopic (2.59 to 6.80 mm) diameters. Nor did we find any influence of pupil decentration, which is still an important preoperative parameter to consider when selecting diffractive multifocal IOLs [35].

This study has some limitations. First, we excluded ocular comorbidities, such as severe dry eye, and any corneal, macular or optic nerve pathology. Therefore, our findings can only be applied to healthy eyes. Second, we correlated many binocular visual outcomes to the average of monocular preoperative variables. Further studies may be useful to understand if our results are confirmed by correlating monocular preoperative variables to monocular postoperative functional outcomes. Third, the non-randomized study design may introduce selection bias, and the single-center nature limits the generalizability of the results beyond the specific study population. Fourth, we did not analyze the possible correlations between the postoperative corneal measurements and the functional performance of the IOL. Fifth, the follow-up was relatively short. Sixth, we did not report the results of the comparison between eyes with toric and non-toric IOLs due to the fact that 9 cases out of 43 had a toric IOL in one eye only and no significant differences were detected between those with (22) and without (12) a toric IOL. Seventh, we did not assess higher-order operations of the cornea at scotopic or mesopic pupil diameters. Finally, the study’s small sample size and the lack of a control group further limit the ability to draw definitive conclusions.

## Conclusions

Both the subjective and objective analyses performed in this study suggest that the visual outcomes for the AcrySof IQ Vivity EDoF-IOL are independent of the patients’ preoperative parameters in healthy eyes. Compared to traditional multifocal IOLs, the preoperative selection of candidates may be of less importance with this IOL. However, attention should be paid to preoperative corneal aberrations and asphericity, which did not lead to visual disturbances, but may be potential sources of halo and reduced contrast sensitivity.

## Data Availability

The datasets used and/or analyzed during the current study are available from the corresponding author upon reasonable request.

## Abbreviations

ACD: Anterior chamber depth
C_w_: Weber contrast units
D: Diopters
DCIVA: Distance-corrected intermediate visual acuity
DCNVA: Distance-corrected near visual acuity
DCVA: Distance-corrected visual acuity
EDoF-IOL: Extended depth-of-focus intraocular lens
HOAs: Higher-order aberrations
LED: Light-emitting diode
MF-IOLs: Multifocal IOLs
MR: Manifest refraction
UDVA: Uncorrected distance visual acuity
